# Blood pressure deficits in acute kidney injury: not all about the mean arterial pressure?

**DOI:** 10.1186/s13054-017-1683-4

**Published:** 2017-05-04

**Authors:** Lui G. Forni, Michael Joannidis

**Affiliations:** 1 Surrey Perioperative Anaesthesia & Critical Care Collaborative Research Group (SPACeR), School of Health Sciences, Faculty of Health & Medical Sciences, University of Surrey, Guildford, UK; 2 Intensive Care Unit, Royal Surrey County Hospital NHS Foundation Trust, Egerton Road, Guildford, GU2 7XX UK; 3 Medical University Innsbruck, Department of General Internal Medicine, Medical ICU, Innsbruck, Austria

**Keywords:** Blood pressure, Acute kidney injury, Blood pressure target, Relative hypotension, Perfusion pressure

## Abstract

Acute kidney injury (AKI) is associated with increased morbidity and mortality. Although there are many causes of AKI, it is known that patients undergoing high-risk surgery are known to be at significant risk. Although much effort has centred on the minimum arterial pressure needed to maintain renal perfusion, this tends to be based on relatively crude measures such as the mean arterial pressure (MAP), which is widely used as an index for the optimal blood pressure. The rationale behind maintaining MAP is to provide adequate organ perfusion, although this is difficult to assess other than by applying crude end-points. Recent studies have examined the progression of AKI as defined by the KDIGO criteria in terms of time-weighted average values for premorbid and within-ICU haemodynamic pressure-related parameters. Although principally performed on patients who had undergone cardiovascular surgery and who were on vasopressor support, some interesting results were obtained suggesting that crude MAP may not be an adequate target in AKI. In patients with AKI progression, greater observed deficits in mean perfusion pressure, diastolic arterial perfusion, and diastolic perfusion pressures were observed. This study may highlight potential modifiable risk factors for the prevention of progression of AKI, and hopefully translate into improved outcomes.

The kidneys account for less than 5% of our total body weight and yet receive about 25% of our cardiac output. It is of no surprise, therefore, that targeting adequate renal perfusion is considered a potential factor for modifying the risk of acute kidney injury (AKI). The risk of AKI varies depending on aetiology, but it is well established that high-risk surgery, in particular cardiovascular surgery, is associated with an increased threat of AKI. Therefore, protective strategies may be instigated prior to, or during, insult in order to minimize this potentially devastating outcome.

The preservation or improvement of renal perfusion may be achieved through increasing cardiac output by fluid resuscitation, inotropic drugs, renal vasodilators, or systemic vasopressors that all “redirect” blood flow to the kidney. The optimal target mean arterial pressure (MAP) remains an area of debate, although several studies have examined this including a multicentre randomised controlled trial (RCT) which randomized patients with septic shock to resuscitation with a MAP target of either 80–85 mmHg or 65–70 mmHg [1]. This study found no difference in mortality or renal end-points such as the incidence of AKI stage 2 or the need for renal replacement therapy (RRT). However, in patients with known chronic hypertension, a higher MAP resulted in both a lower incidence of AKI stage 2 and less RRT but, importantly, there was no effect on mortality. Lowering systolic pressure has been studied in isolation in patients with acute cerebral haemorrhage randomized to a systolic blood pressure target of 110–139 mmHg or 140–179 mmHg. The primary end-point (death or disability) was not different between groups, although the rate of serious renal adverse events was higher in the lower blood pressure target group [2]. In terms of potential treatment, norepinephrine is the most commonly used vasopressor in patients with vasodilatory shock although vasopressin or the analogue terlipressin may have a role in norepinephrine refractory shock [3, 4]. Exogenous vasopressin has vasoconstrictive and anti-diuretic properties and may increase glomerular filtration by preferential post-glomerular vasoconstriction and hence may play a role [5].

To date, most studies have examined the MAP rather than specific components of the derived MAP. However, Saito and colleagues have examined patients on vasopressors who had undergone cardiovascular surgery with regard to the progression of AKI as defined by the KDIGO criteria [6]. They measured time-weighted average values for premorbid and within-ICU haemodynamic pressure-related parameters including systolic arterial pressure (SAP), diastolic arterial pressure (DAP), and MAP, and, in addition, central venous pressure (CVP; initially measured through inferior vena cava parameters (diameter and collapse) derived from outpatient echocardiography examinations and subsequently from direct measurements on the ICU). The mean perfusion pressure (MPP) and diastolic perfusion pressure (DPP) were calculated, with the differences between premorbid and ICU values determined and the calculated deficits in those values computed. This follows on from an observational cohort study that investigated mean deficits between premorbid and actual mean perfusion pressure in vasopressor-dependent ICU patients and identified “relative hypotension”, reporting an association between the reduced MPP and the development of AKI [7]. Both these studies focus once again on the pivotal role of renal blood flow in the development of AKI under certain conditions, particularly where renal hypoxia (at whatever level) is thought to be significant.

When considering alterations to the systemic blood pressure and effects on the renal circulation, however, several features must be borne in mind. Firstly, the renal circulation can be viewed as two circulations in series: that to the glomerular capillaries through the afferent arteriolar system, and that to the tubular network via the efferent arterioles. Secondly, in order to maintain adequate pressure within the glomerular and distal capillaries, these systems are under intimate local control. This balance between the afferent and efferent systems, termed auto-regulation, is a fundamental component of renal function.

Autoregulation stabilizes renal blood flow (RBF) and the glomerular filtration rate (GFR) during variations in renal perfusion pressure (RPP) over a defined range, implying that renal vascular resistance (RVR) changes in proportion to the RPP. The major pre-glomerular resistance vessels whose tone mediates most of this pressure-induced auto-regulation includes the afferent and cortical radial arteries. The efferent arterioles only play a minor role in RBF auto-regulation, principally under conditions of low RPP. Auto-regulation occurs through two major mechanisms: the myogenic response and the macula densa tubuloglomerular feedback (TGF) response, which are outlined in Fig. 1. The myogenic response reflects afferent arteriolar tone in response to changes in intraluminal pressure, whereas TGF is a more delayed response activated by tubular electrolyte delivery and re-absorption and, as such, associates the GFR with NaCl delivery. Consequently, TGF integrates *both* tubular and vascular function, and together these mechanisms adjust the tone of the pre-glomerular vessels in the face of a changing RPP. Local mediators also govern these responses; these include angiotensin II, arachidonate metabolites, and NO. Thus, auto-regulation is intrinsic to the kidney and largely independent of extrinsic nerves or circulating hormones [8]. Such auto-regulation is not unique, and in many organs blood flow is regulated to satisfy the metabolic demands. However, what is different in the kidney is that the renal metabolic work is a *function* of RBF and is specifically dependent on the GFR and the amount of sodium that is to be actively re-absorbed [9, 10]. Therefore, the higher the blood flow the higher the metabolic demands. Given that under normal conditions renal sympathetic tone is low, a relatively high resting blood flow rate is observed within a relatively low resistance system. Interestingly, these flow waveforms observed are similar to that seen in the brain: both a relatively high diastolic pressure and mean flow velocity.

**Fig. 1:**
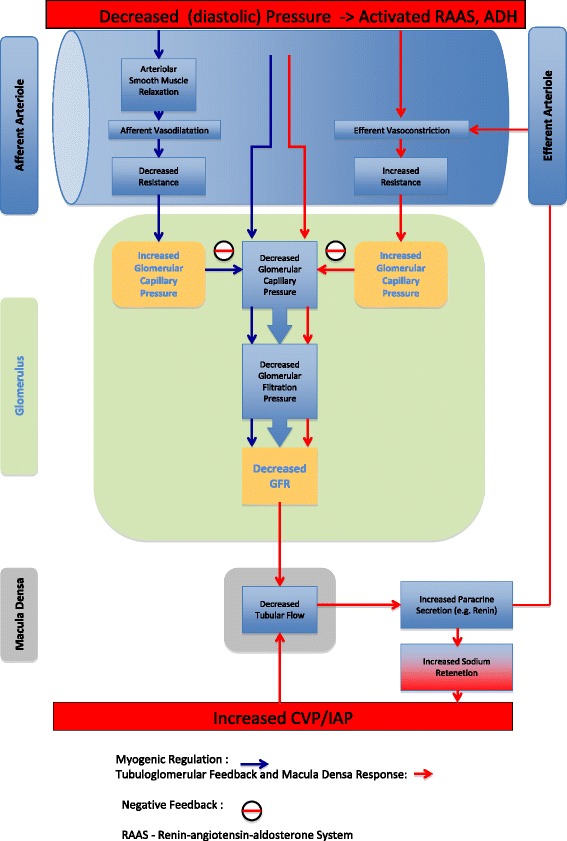
Myogenic regulation is indicated by *blue arrows*, and the macula densa tubuloglomerular feedback response is indicated by *red arrows*. Negative feedback is indicated by a red line in a black circle. *ADH* anti-diuretic hormone, *CVP* central venous pressure, *GFR* glomerular filtration rate, *IAP* intra-abdominal pressure, *RAAS* renin-angiotensin-aldosterone system

So, what did Saito and colleagues observe? In terms of measured haemodynamic parameters there was no difference in percentage SAP or MAP deficit between the AKI+ (AKI progression group) and the AKI– (those who did not progress). However, DAP, MPP, and DPP were all significantly different, with the greatest difference observed in the diastolic perfusion pressure (*p* = 0.027, 0.023, and 0.002, respectively). The AKI+ group had consistently greater deficits of all three perfusion pressures in the first 24 h of vasopressor support compared with the AKI– group, with 21.7% and 23.8% of the percentage MPP and DPP deficits, respectively, due to an increase in CVP, with no difference between the AKI+ and AKI– groups. Also of note is that the median MAP of 74 was similar in both groups. Although this study has several limitations (small sample, higher baseline creatinine and MPP in those that progressed, single-centre etc., all of which the authors concede to), the results are still intriguing. The suggestion that DPP or indeed MPP may be viewed as haemodynamic targets in this high-risk group would fit with the recent consensus regarding blood pressure targets in ICU patients being targeted to the individual, with evidence from studies in sepsis supporting this view [11, 12].

The tailored approach to optimal blood pressure control has some credence. A recent study using ultrasound-tagged near infrared spectroscopy (UT-NIRS) during cardiopulmonary bypass (CPB) and in the first 3 h after surgery in the ICU examined real-time monitoring of cerebral blood flow (CBF). This allowed derivation of the correlation flow index (CFx) which was calculated as a moving, linear correlation coefficient between cerebral flow index measured using UT-NIRS and MAP [13]. This is similar to the mean velocity index derived from transcranial Doppler [14]. Optimal blood pressure was defined as the MAP with the lowest CFx, given that when the MAP is outside the auto-regulation range CFx becomes more positive as CBF is dependent on changes in blood pressure. They observed AKI in just over 27% of patients (predominantly KDIGO 1). Multivariate logistic regression for the development of AKI showed that the blood pressure excursion below the optimal blood pressure during CPB and in the ICU was independently associated with CSA-AKI after adjusting for possible confounding variables including total CPB time, total cross-clamp time, pre-operative estimated GFR, and surgery other than isolated coronary artery bypass grafting (CABG).

In conclusion, we may be entering an era where the application of non-individualised haemodynamic targets based on empiric blood pressure may be a practice of the past. In similar fashion to the use of intra-operative monitoring of volume response to guide fluid therapy, real-time monitoring of blood flow coupled with attention to previously ignored parameters may well guide intra-operative blood pressure management as well as that on the ICU. Hopefully this will translate to improved outcomes.

## Abbreviations

AKI: Acute kidney injury
CBF: Cerebral blood flow
CFx: Correlation flow index
CPB: Cardiopulmonary bypass
CVP: Central venous pressure
DAP: Diastolic arterial pressure
DPP: Diastolic perfusion pressure
GFR: Glomerular filtration rate
MAP: Mean arterial pressure
MPP: Mean perfusion pressure
RBF: Renal blood flow
RPP: Renal perfusion pressure
RRT: Renal replacement therapy
SAP: Systolic arterial pressure
TGF: Tubuloglomerular feedback
UT-NIRS: Ultrasound-tagged near infrared spectroscopy
